# Simultaneous extraction, optimization, and analysis of flavonoids and polyphenols from peach and pumpkin extracts using a TLC-densitometric method

**DOI:** 10.1186/s13065-015-0113-4

**Published:** 2015-06-20

**Authors:** Ammar Altemimi, Dennis G. Watson, Mary Kinsel, David A. Lightfoot

**Affiliations:** Department of Plant, Soil and Agricultural Systems, Southern Illinois University, Carbondale, IL 62901 USA; Department of Food Science, College of Agriculture, University of Basrah, Basrah, 61004 Iraq; SIUC Mass Spectrometry Facility, Department of Chemistry and Biochemistry, SIUC, Carbondale, IL 62901 USA

**Keywords:** Ellagic acid, Rutin, Myricetin, Ultrasound, TLC, Densitometry, Mass spectrometry

## Abstract

**Background:**

The use of medicinal plants has been reported throughout human history. In the fight against illnesses, medicinal plants represent the primary health care system for 60 % of the world’s population. Flavonoids are polyphenolic compounds with active anti-microbial properties; they are produced in plants as pigments. Quercetin, myricetin, and rutin are among the most well-known and prevalent flavonoids in plants, with an antioxidant activity capable of decreasing the oxidation of low density lipoproteins [LDLs]. To date, this research is the first of its kind to employ a coupled thin-layer chromatography (TLC) and a densitometric quantification method with a Box-Behnken design (BBD) response surface methodology (RSM) for optimization of ultrasonic-assisted extraction and determination of rutin and quercetin from peach and ellagic acid and myricetin from pumpkin fruits.

**Results:**

The effect of process variables (extraction temperature (°C), extraction power (%) and extraction time (min)) on ultrasound-assisted extraction (UAE) were examined by using BBD and RSM. TLC followed by Quantity-One™ (BioRad) image analysis as a simple and rapid method was used for identification and quantification of the compounds in complex mixtures. The results were consistent under optimal conditions among the experimental values and their predicted values. A mass spectrometry (MALDI-TOF MS) technique was also used to confirm the identity of the natural products in the TLC spots resolved.

**Conclusion:**

The results show that the coupled TLC-densitometric methods & BBD can be a very powerful approach to qualitative and quantitative analysis of; rutin and quercetin from peach extracts; and ellagic acid and myricetin contents from pumpkin extracts.

## Introduction

The use of medicinal plants has been reported throughout human history [1]. In the fight against illnesses, medicinal plants represent the primary health care system for 60 % of the world’s population. With the advent of chemistry, modern pharmacotherapy has depended more on synthetic drugs, however due to raised safety concerns and lower drug efficiency there is a growing interest to show direct evidence of the crucial role of natural secondary metabolites [2–4].

Flavonoids are polyphenolic compounds with active phytoalexins (anti-microbial) properties; they are produced in plants as pigments [5]. Quercetin, myricetin, and rutin are among the most well-known and prevalent flavonoids in plants, with an antioxidant activity capable of decreasing low density lipoproteins [LDL] oxidation [6]. Different types of hydro-alcoholic mixtures have been used to extract flavonoid from the plant material [7, 8]. Methanol or ethanol was employed depending on the targeted compounds [9]. Extraction techniques for flavonoids are typically called traditional and modern methods [10]. Maceration, reflux, percolation, and Soxhlet extraction are within the lists of traditional methods, which have been significantly improved by automation [11]. Currently the focus has been on modern methods that were developed to be more efficient, faster, and with lower consumption of organic solvent [10, 12, 13]. Some of the modern methods are ultrasound assisted extraction (UAE), microwave assisted extraction (MAE), pressurized liquid extraction (PLE), and supercritical fluid extraction (SFE) [14, 15].

Many methods have been used to isolate and measure the activity of antioxidant compounds [16]. Recently, thin layer chromatography (TLC) analysis of flavonoids in plant and animal samples was used for studying the application of scanning densitometry in quantification of flavonoids [17]. TLC is widely used because it is relatively simple, rapid, inexpensive, and accurate method for chemical identification when coupled with mass spectrometry (MS) [18]. TLC combined with densitometry and image analysis can have the ability to measure medicinal plant components [19]. Densitometry can be used to measure the differences among absorbance or fluorescence signals between a separated zone and the empty plate background across a range of wavelengths [20]. Image analysis methods are used to compare the spot color intensity with the plate color background. The peak area of the test spots are compared with data from calibration standards chromatographed on the same plate [21, 22].

Several techniques have been used in the past for determining plant extracts, including TLC [23]; GC-MS [24]; and HPLC [25]. There are no known reports that described a coupled TLC-densitometry method for quantitative determination of; ellagic acid and myricetin from pumpkin; or quercetin and rutin from peach. This study employed a coupled TLC densitometric method and Box-Behnken design (BBD) with response surface methodology (RSM) for optimization of UAE. Isolated products identities were confirmed by using matrix-assisted laser desorption/ionization time-off-light MS (MALDI-TOF MS) analysis.

### Experimental

#### Plant material

All crops were grown on bare soil, a silt loam typical of southern Illinois. Fresh peaches (Red Haven) and pumpkins (Libbys Select) were harvested from several plants selected at random within a field at the Horticulture Research Center farm on Rowden Road near Southern Illinois University (Carbondale, IL). Peaches and pumpkins were grown according to conventional commercial methods for southern Illinois. Synthetic nutrients and pesticides were applied according to recommendations for peach and pumpkin production in southern Illinois. The samples were provided by Dr. Alan Walter (Department of Plant, Soil and Agricultural Systems, College of Agricultural Sciences, Southern Illinois University, USA). The fruits were cleaned and sliced into small pieces and crushed in a blender, and then sealed and stored in plastic bags in home refrigerator (−18 °C) for five days before freeze-drying.

### Ultrasonic assisted extraction (UAE)

An Elmasonic P30 (P30) ultrasonic device with heated water bath (Elma Hans Schmidbauer GMBH, Singen, Germany) set at 37 kHz was used for this study. User adjustable controls were heated bath temperature and power setting as a percentage of full power (30–100 %). The standard ultrasonic mode was used. The manufacturer rated the P30 with an effective power rating of 120 W. The P30 had a proprietary algorithm to adjust power based on the impedance of the system, resulting in the effective power rating. For a specific power setting, samples experienced the same degree of cavitation regardless of the load in the tank. For all treatments, the bath of the P30 contained 1.7 L of water before the treatment containers were added. Ultrasonic power was expressed as W/cm^2^, based on the power setting as a percentage of rated power and the volume of the bath solution.

Although numerous variables my affect a process, identifying and controlling each variable with small contributions is practically impossible, therefore, variables were selected with known major effects [26]. The prior work of Altemimi et al. [27, 28] with the same ultrasonic equipment was used as a guide and selected variables were bath temperatures of 30 °C, 40 °C, and 50 °C; power level settings of 30 %, 50 %, and 70 %; and ultrasonic duration of 10 min, 20 min, and 30 min. The ultrasonic bath temperature was controlled by coupling with a cooling system using a cooling coil (Fisher Scientific Inc. St Louis USA) and water pump (model HJ-111, submersible pump, flow rate 250 L/h, Sunsun Inc., Zhejiang, China). Coupled heating and cooling helped to maintain temperatures that were evenly distributed across the ultrasonic water bath. Based on the manufacturer’s effective power rating, the ultrasonic power for the three power settings inside the extract containers was 21 W/cm^2^, 35 W/cm^2^, and 49 W/cm^2^, respectively. A calorimetric method was used to independently verify the power settings [29].

Ten grams of the lyophilized samples were weighed and 100 mL of methanol were added to the samples in a 200 mL glass flask. Each flask was placed in the P30 and treated. After the samples had been exposed to ultrasound waves, the upper layer was filtered (Whatman no. 1) and placed in a rotary evaporator under vacuum at 40 °C to remove solvent.

### Experimental design

The effects of three independent variables of temperature, power, and time to optimize the extracted amount of compounds were investigated by using a BBD for RSM. The coded values of the experimental factors and settings for the experimental design were summarized in Table 1. The 17 ultrasonic treatments were completed in random order. The experimental data were analyzed with multiple regressions to fit the quadratic polynomial model in Eq. 1.

1 $$ Y={b}_0{\displaystyle {\sum}_{\mathrm{i}=1}^3{b}_{\mathrm{i}}{X}_{\mathrm{i}}}+{\displaystyle {\sum}_{\mathrm{i}=1}^3{b}_{\mathrm{i}\mathrm{i}}}{X^2}_{\mathrm{i}}+{\displaystyle {\sum}_{\mathrm{i}\ne \mathrm{j}=1}^3{b}_{\mathrm{i}\mathrm{i}}{X}_{\mathrm{i}}{X}_j} $$

**Table 1:** Combinations of temperature, power and time with their coded level terms in parentheses terms obtained from RSM^a^ and the observed values of rutin, quercetin, ellagic acid, and myricetin

Run	Factor 1: A temperature °C	Factor 2: B power %	Factor 3: C time min	Rutin μg/g	Quercetin μg/g	Ellagic acid μg/g	Myricetin μg/g
1	40 (0)	30 (−1)	10 (−1)	2.65	2.54	3	2.96
2	50 (1)	50 (0)	10 (−1)	2.56	2.44	2.88	2.84
3	40 (0)	70 (1)	10 (−1)	2.67	2.55	2.98	2.94
4	30 (−1)	50 (0)	30 (1)	2.72	2.6	2.69	2.65
5	40 (0)	50 (0)	20 (0)	2.90	2.8	2.99	2.95
6	40 (0)	70 (1)	30 (1)	2.79	2.66	2.83	2.79
7	50 (1)	50 (0)	30 (1)	2.75	2.63	2.89	2.85
8	30 (−1)	30 (−1)	20 (0)	2.57	2.45	2.93	2.89
9	40 (0)	50 (0)	20 (0)	2.89	2.77	3.03	2.98
10	50 (1)	70 (1)	20 (0)	2.72	2.6	2.98	2.94
11	30 (−1)	50 (0)	10 (−1)	2.54	2.42	2.85	2.81
12	30 (−1)	70 (1)	20 (0)	2.62	2.5	2.79	2.75
13	40 (0)	50 (0)	20 (0)	2.89	2.78	3.02	2.97
14	50 (1)	30 (−1)	20 (0)	2.68	2.56	2.88	2.84
15	40 (0)	50 (0)	20 (0)	2.88	2.76	2.95	2.91
16	40 (0)	30 (−1)	30 (1)	2.71	2.59	2.95	2.91
17	40 (0)	50 (0)	20 (0)	2.85	2.73	3.02	2.95

^a^The results were obtained with the Design Expert 9.0 software

Where Y is the predicted response; b_0_ is the intercept; b_1_, b_2_ and b_3_ are the linear coefficients of temperature (X_1_), power (*X*_2_) and time (X_3_), respectively; b_11_, b_22_ and b_33_ are the squared coefficient of temperature of sonication, power and time, respectively; b_12_, b_13_ and b_23_ are the interaction coefficients of temperature of sonication, power and time, respectively. The settings of the independent variables were represented as X_i_ to X_j_.

### Thin layer chromatography chemical screening

**Fig. 1:**
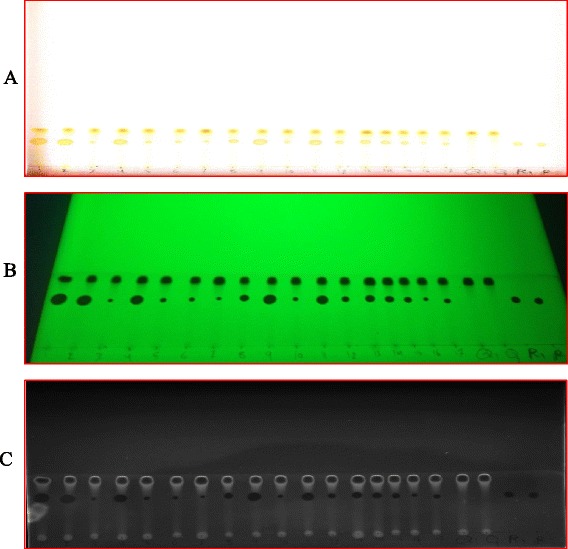
The chromatographic separation of quercetin (Q) and Rutin (R): (**a**) real image; (**b**) UV image at at 254 nm; and (**c**) grey scale image quantity one software. Spots: 1 to 17 for samples extracts

**Fig. 2:**
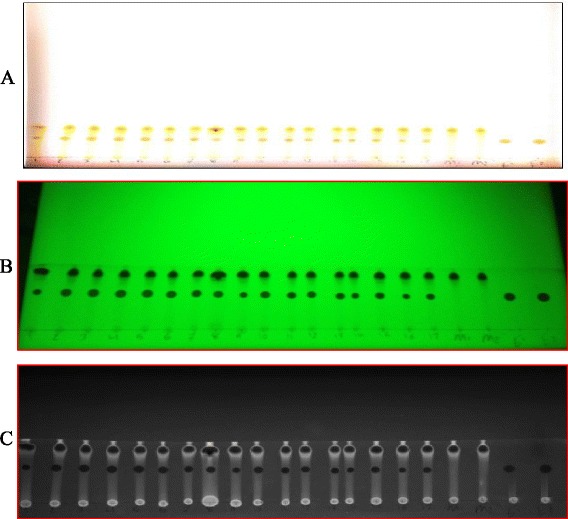
The chromatographic separation of myricetin (M) and ellagic acid (E): (**a**) real image; (**b**) UV image at at 254 nm; and (**c**) grey scale image by quantity one software. Spots: 1 to 17 for samples extracts

The glass TLC plates were 20 cm by 20 cm and pre-coated with silica gel 60 F254 (E. Merck/Millipore, Billerica, MA) (0.2 mm thickness). The following solvents were screened to determine the best separation compound for the TLC technique: 1) ethyl acetate 5: acetone 4 (v/v), 2) hexane 10: chloroform 10 (v/v), and 3) ethyl acetate10: formic acid 2: water 3 (v/v). The TLC plate was placed into oven at 110 °C for 20–30 min to be completely dried. Each of the solvents was evaluated by mixing and placing 100 mL into a rectangular chromatography glass tank with ground edges. The glass tank was covered with a glass lid and solvents were allowed to saturate for 30–40 min before use. Two μL of each crude extract were added by syringe to a different TLC plate. The crude extracts were placed in a drop shape for identification and spread of the separated compounds according to Harbone [30]. Flavonoids have a weak natural fluorescence characterization and must be enhanced during separation on chromatography plates. The flavonoids fluorescence was enhanced by spraying the TLC plates with different complex agents. The most common complex agent used to increase the flavonoids fluorescence was the diphenyl-boric acid 2-amino ethyl ester (DPBA) [31]. Images of the TLC plates were analyzed using Quantity One™ densitometry software (Bio-Rad, Hercules, CA). The compounds in the samples were quantified by comparing density of the peaks and their areas (expressed as intensity per mm^2^) from the samples with those from standard solutions of rutin, qurecetin, ellagic acid, and myricetin on the same plate. The best separation was obtained by ethyl acetate10: formic acid 2: water 3 (v/v) [32]. The software evaluated the area of separated spots by comparing the spot color intensity to the color of the TLC plate background. It was essential to chromatograph the standards on the same plates to compensate for slight variations among the different plates (Figs. 1 and 2). The software generated a final chromatogram image that allowed the quantitative evaluation of the TLC separation by densitometry.

### Preparation of calibration curves for rutin and quercetin from peach extracts and ellagic acid and myricetin frompumpkin extracts

All chemicals used in the experiments were analytical grade reference standard compounds. Rutin (R) (purity 98 %) was procured from Indofine Chemical Company (Hillsborough Township, NJ, USA) and quercetin (Q) (purity 99.32 %) was purchased from MP Biomedicals LLC (Santa Ana, CA, USA). ellagic acid (E) (purity 99 %) was procured from MP Biomedicals LLC (Santa Ana, CA, USA). and myricetin (M) (purity 98 %) was purchased from Alfa Aesar, MA, USA. The stock solution of rutin (70 μg.μL^−1^) was prepared in methanol. Different volumes of stock solution 2, 4, 6, 8, 10, and 12 μL, were spotted on the TLC plate to obtain concentrations of 140, 280, 420, 560, 700, and 840 μg spot^−1^ of rutin, respectively. The stock solution of quercetin (80 μg.μL^−1^) was prepared in methanol. Different volumes of the stock solution 2, 4, 6, 8, 10, and 12 μL, were spotted on the TLC plate to obtain concentrations of 160, 320, 480, 690, 800, and 960 μg spot^−1^ of quercetin, respectively. Different volumes of stock solution 2, 4, 6, 8, 10, and 12 μL, were spotted on the TLC plate to obtain concentrations of 150, 300, 450, 600, 750, and 900 μg spot^−1^ of ellagic acid, respectively. The stock solution of myricetin (70 μg.μL^−1^) was prepared in methanol. Different volumes of the stock solution 2, 4, 6, 8, 10, and 12 μL, were spotted on the TLC plate to obtain concentrations of 140, 280, 420, 560,700, and 840 μg spot^−1^ of myricetin, respectively. These spots of the reference compounds were used to determine the calibration curves for the TLC-densitometry. The calibration curves were used by Quantity One™ software to generate accurate quantification of rutin, quercetin, ellagic acid, and myricetin in the experimental samples.

### Simultaneous quantification of rutin and quercetin from peach extracts and ellagic acid and myricetin from pumpkin extracts

Two μL of each of the 17 plants extracts were applied on a TLC plate. The plate was developed and scanned as described in the TLC chemical screening process. The peak areas were recorded and the amounts of rutin and quercetin from peach extracts and ellagic acid and myricetin from pumpkin extracts were calculated using the respective calibration curves.

### Diagnostic checking of RSM model and validity testing

Design-Expert™ software (version 9) was used to analyze the experimental results of the response surface design (State-Ease lnc. Minneapolis, MN, USA). *P*-values less than 0.05 were used to determine statistical significance of differences. Independent variables of extraction temperature, ultrasonic power, and extraction time were simultaneously optimized using RSM. Subsequently the output for each isolated compound was measured from peach and pumpkin extracts under the optimum ultrasonic conditions. The ultrasonic experiments using the optimum conditions were replicated three times and the results were compared with the predicted values for validation of the model.

### Mass spectrometric analysis

The confirmation of each TLC spot identity was achieved using time-of-flight mass spectrometry. Each TLC spot of interest was excised and the compound was extracted into methanol. The freshly extracted compounds were then prepared for either matrix – assisted laser desorption ionization (MALDI) or laser desorption ionization (LDI). Certified standards of quercetin, rutin, ellagic acid, and myricetin were analyzed in tandem to confirm the identity of each compound.

A 1 − μL aliquot of TLC Spot Q, R, and M methanol extracts were spotted separately with 1 − μL of a MALDI matrix solution of α - cyano-4-hydroxycinnamic acid (αCHCA, 5 mg/mL αCHCA in 50:50 (v:v) acetonitrile: 0.1 % (v/v) trifluoroacetic acid in water). A 1 μL aliquot of TLC Spot E methanol extract was also spotted on the stainless steel sample plate with no MALDI matrix. The TLC Spots Q, R, M, and E were allowed to dry at room temperature. The stainless steel sample plate containing the dried MALDI and LDI samples was inserted into Bruker Daltonics (Billerica, MA, USA) MicroFlexLR time-of-flight mass spectrometer. The samples were irradiated with a pulsed nitrogen laser and the positive ion signal was recorded in the mass-to-charge (*m/z*) region of 20 to 1000. Each mass spectrum consisted of an average of 1000 laser shots.

## Results and discussion

### Chromatographic separation and image analysis software

TLC-densitometry coupled with image analysis detection was evaluated for the quantitative determination of induced flavonoids. According to the Figs. 1 and 2, the images allowed a visual evaluation of the flavonoids and polyphenolic acids (yellow-orange fluorescence) [33]. The method was suitable for rapid quantification of rutin and qurecetin in peach extracts and ellagic acid and myricetin in pumpkin extracts. It required less time for sample preparation and quantification compared to HPLC. These findings were in reasonable agreement with Nikolova et al. [22] and Naşcu-Briciu et al. [10] who found that TLC- densitometric analysis with image analysis software was complementary to the photodensitometric methods.

### Fitting the models

The preliminary experiments were very advantageous in order to screen and choose the levels of independent variables for peach and pumpkin extracts. The experimental design for Box-Behnken and corresponding response data are presented in Table 1. According to the results in Table 1, the quadratic polynomial model was assigned for multiple regression analysis. The contribution of the quadratic model within regression coefficients analysis and the analysis of variance (ANOVA) are shown in Tables 2 and 3 [34]. In general, the variation in the data around the fitted model was examined using lack of fit test for the model [35]. Lack of fit must not be significant (*p* > 0.05) for an appropriate model.

**Table 2:** Analysis of variance results for the regression (peach)

Source	Degree of freedom	Sum of square	Mean square	F-value	*p*-value
Rutin					
Model	9	0.2400	0.027	17.41	0.0005
X_1_	1	0.0084	0.0084	5.52	0.0512
X_2_	1	0.0045	0.0045	2.95	0.1298
X_3_	1	0.0378	0.0378	24.68	0.0016
X_1_X_2_	1	2.500	2.500	0.016	0.9019
X_1_X_3_	1	2.500	2.500	0.016	0.9019
X_2_X_3_	1	0.0009	0.0009	0.59	0.4685
${\mathrm{X}}_1^2$	1	0.0979	0.0979	63.91	<0.0001
${\mathrm{X}}_2^2$	1	0.0341	0.0341	22.26	0.0022
${\mathrm{X}}_3^2$	1	0.038	0.038	24.80	0.0016
Lack of fit	3	0.007725	0.002575	3.43	0.1322
Quercetin					
Model	9	0.2336	0.026	12.86	0.0014
X_1_	1	0.0024	0.0024	1.21	0.3070
X_2_	1	0.0097	0.0097	4.85	0.0634
X_3_	1	0.0024	0.0024	1.21	0.3070
X_1_X_2_	1	0.0006	0.0006	0.31	0.5952
X_1_X_3_	1	0.0042	0.0042	2.09	0.1912
X_2_X_3_	1	0.0306	0.0306	15.17	0.0059
${\mathrm{X}}_1^2$	1	0.1047	0.1047	51.91	0.0002
${\mathrm{X}}_2^2$	1	0.0254	0.0254	12.61	0.0093
${\mathrm{X}}_3^2$	1	0.0362	0.0362	17.94	0.0039
Lack of fit	3	0.0087	0.0029	3.88	0.1117

**Table 3:** Analysis of variance results for the regression (pumpkin)

Source	Degree of freedom	Sum of square	Mean square	F-value	*p*-value
Ellagic acid					
Model	9	0.1341	0.0150	14.30	0.0010
X_1_	1	0.0171	0.0171	16.42	0.0049
X_2_	1	0.0040	0.0040	3.89	0.0893
X_3_	1	0.0153	0.0153	14.69	0.0064
X_1_X_2_	1	0.0144	0.0144	13.82	0.0075
X_1_X_3_	1	0.0072	0.0072	6.93	0.0338
X_2_X_3_	1	0.0025	0.0025	2.40	0.1653
${\mathrm{X}}_1^2$	1	0.0516	0.0516	49.56	0.0002
${\mathrm{X}}_2^2$	1	1.2894	1.2894	0.012	0.9146
${\mathrm{X}}_3^2$	1	0.0182	0.0182	17.47	0.0041
Lack of fit	3	0.0025	0.00085	0.7274	0.586
Myricetin					
Model	9	0.1277	0.0141	15.30	0.0008
X_1_	1	0.0171	0.0171	18.44	0.0036
X_2_	1	0.00405	0.00405	4.36	0.0751
X_3_	1	0.01531	0.01531	16.50	0.0048
X_1_X_2_	1	0.0144	0.0144	15.52	0.0056
X_1_X_3_	1	0.0072	0.0072	7.79	0.0269
X_2_X_3_	1	0.0025	0.0025	2.69	0.1447
${\mathrm{X}}_1^2$	1	0.0479	0.0479	51.71	0.0002
${\mathrm{X}}_2^2$	1	0.0001	0.0001	0.15	0.7100
${\mathrm{X}}_3^2$	1	0.0160	0.0160	17.30	0.0042
Lack of fit	3	0.0025	0.00085	0.88	0.5244

### Effect of ultrasonic parameters on rutin and quercetin contents of peach and analysis of response surfaces

ANOVA analysis and regression coefficients (Table 2) were obtained in order to test the fitted quadratic surface models for rutin and quercetin content in peach extracts. For rutin content (μg/g of dry matter), the linear parameter (time) was significant and interaction parameters (temp*power, temp* time, time*power) were not significant (*p* > 0.05), whereas all quadratic parameters were significant (*p* < 0.05). In quercetin content (μg/g of dry matter), the interaction parameter (time*power) was significant at the level of *p* < 0.001 and the parameters (temp*power, temp*time) were not significant (*p* > 0.05) while all quadratic parameters were significant at the level of *p* < 0.05. F-values for lack-of-fit were 3.43 and 3.88 for rutin and quercetin, respectively. The lack-of-fit was not significant (*p* > 0.05). The R^2^ of the models for rutin and quercetin content were 0.9572 and 0.9528, respectively. Moreover, the coefficients of variation (CV) were 1.43 and 1.57 for rutin and quercetin, respectively. Experimental results were predicted with good accuracy when a low coefficient of variation (CV) was obtained.

Three-dimensional plots was used to better understanding the relationship between independent and dependent variables, then the following quadratic polynomial model equations (2, 3) were assigned to generate the contour plots:

2 $$ \begin{array}{l}\mathrm{Rutin}=2.89+0.0325*{X}_1+0.02375*{X}_2+0.06875*{X}_3-0.0025*{X}_1{X}_2+0.0025*{X}_1{X}_3+\\ {}0.015*{X}_2{X}_3-0.1525*{X}_1^2-0.09*{X}_2^2-0.095*{X}_3^2\end{array} $$

3 $$ \begin{array}{l}\mathrm{Quercetin}=2.77+0.0325*{X}_1+0.02125*{X}_2+0.06625*{X}_3-0.0024*{X}_1{X}_2+0.0025*{X}_1{X}_3+\\ {}0.015*{X}_2{X}_3-0.1525*{X}_1^2-0.09*{X}_2^2-0.095*{X}_3^2\end{array} $$

**Fig. 3:**
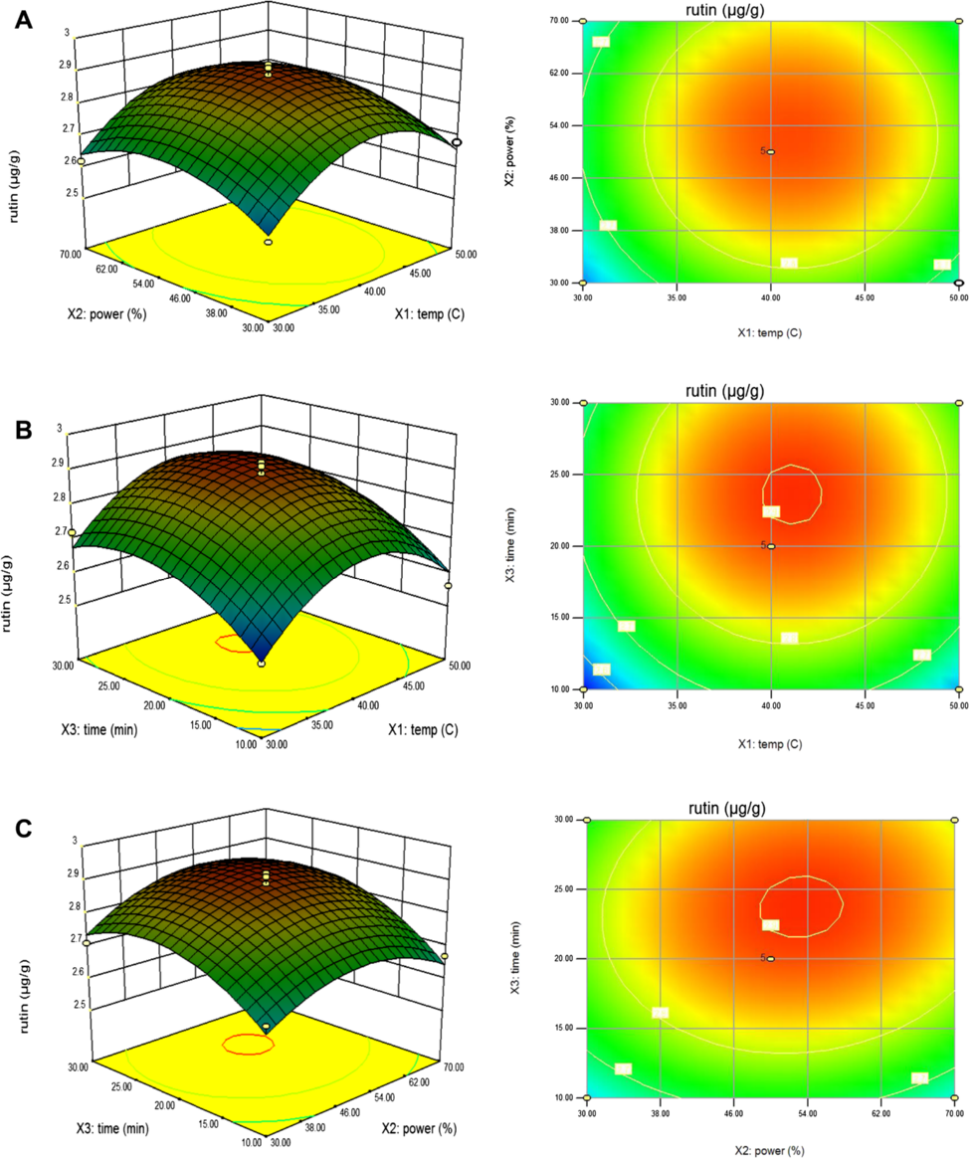
Response surface model plot showing the effects of of independent variables on rutin content. Panel (**a**) temperature and power. Panel (**b**) temperature and time. Panel (**c**) power and time

**Fig. 4:**
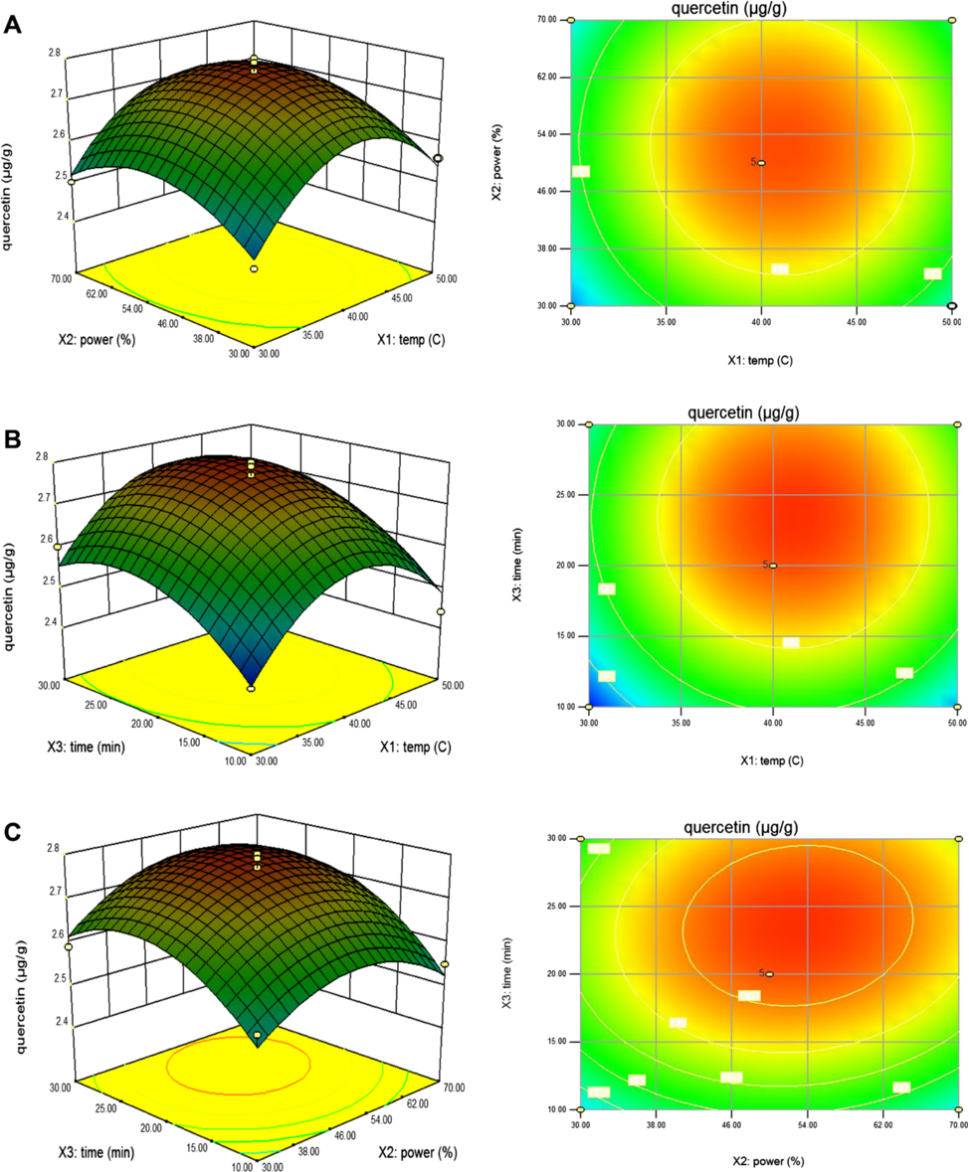
Response surface model plot showing the effects of of independent variables on quercetin content. Panel (**a**) temperature and power. Panel (**b**) temperature and time. Panel (**c**) power and time

The effects of parameter variables (ultrasonic temperature, power, and extraction time) and their interactions on rutin and quercetin contents in peach were studied. The third variable was assigned to be constant at the intermediate setting while surface plots of three-dimensions were shown by two independent variables. As shown in Fig. 3a, with increase extraction temperature from 30 °C to 41.08 °C, the extraction amount of rutin quickly increased and reached the maximum value at 0 level of extraction time in the fixed extraction power of 53.24 %. However, with the increase of extraction temperature from 41.08 °C to 50 °C, the amount of rutin quickly decreased. This result confirmed that higher temperature can enhance the solubility of the solute thereby increases the yield of flavonoids. But, at the same time, increasing temperature can reduce the solvent density and consequently decreases the yield of total flavonoids. Therefore, the increase in temperature could have either a positive or a negative effect [36]. This finding was in agreement with Zhong [37] who reported that the thermal degradation of flavonoids and the decrease of number of acoustic cavitation bubbles were caused to decrease the amount of rutin. Figure 3b shows the effect of the interaction of extraction temperature and extraction time on the rutin content at a fixed extraction power of 0 level. Maximum rutin content was obtained at 41.08 °C and then decreased slightly by increasing extraction temperature to 50 °C in the fixed extraction time of 23.77 min. Figure 3c shows the effect of the interaction of extraction power and extraction time on the rutin content at a fixed extraction temperature of 0 level. Maximum rutin content was obtained at the highest extraction time in the fixed extraction power of 53.24 %. Moreover, the results found that extraction time (X_3_) was the most significant factor affecting the responses at the level of *p* < 0.01. Figure 4a shows the effect of the interaction of extraction temperature and extraction power on the quercetin content at a fixed extraction time of 0 level.

Maximum quercetin content was obtained at the lowest extraction temperature and reached the maximum value at 41.11 °C of extraction temperature in the fixed extraction power of 52.92 %. Figure 4b shows the effect of the interaction of extraction temperature and extraction time on the quercetin content at a fixed extraction power of 0 level. Maximum quercetin content was also obtained at the lowest extraction temperature and then decreased slightly by increasing extraction temperature to 50 °C in the fixed extraction time of 23.61 min. The decrease may be explained by oxidation and degradation of flavonoids due to sonication process with both highest extraction temperature and longest extraction time [38]. Figure 4c shows the effect of the interaction of extraction power and extraction time on the quercetin content at a fixed extraction temperature of 0 level. Maximum quercetin content was obtained at 52.92 % of extraction power in the fixed extraction time of 23.61 min.

### Effect of ultrasonic parameters on ellagic acid and myricetin contents of pumpkin and analysis of response surfaces

Table 3 lists the analysis of variance of the fitted quadratic polynomial model for ellagic acid and myricetin contents in pumpkin extracts. For ellagic acid content (μg/g of dry matter), the linear parameters (temp, time) were significant; interaction parameter (temp* power) and (temp*time) were significant (*p* < 0.05) while all quadratic parameters were significant at the level of *p* < 0.05. In myricetin content (μg/g of dry matter), the linear parameters (temp, time) were significant; the interaction parameters (temp*power, temp*time) were significant at the level of *p* < 0.001 and (power* time) was not significant (*p* > 0.05) while quadratic parameters $\left({\mathrm{X}}_1^2,{\mathrm{X}}_3^2\right)$ were significant at the level of *p* < 0.05. The F-value of 14.30, 15.30 of ellagic acid and myricetin contents respectively implied the model was significant. The lack-of-fit F-value of 0.7274 and 0.88 of ellagic acid and myricetin contents respectively reflects that the lack-of-fit was not significant. The R^2^ of the models for ellagic acid and myricetin contents were 0.9484 and 0.9516, respectively. Moreover, the coefficients of variation (CV) were 1.10 and 1.06 for ellagic acid and myricetin contents, respectively.

Response surface models were used according to the following quadratic polynomial model equations (4, 5) in order to study the effects of parameter variables (ultrasonic temperature, power, and extraction time) and their interactions on ellagic acid and myricetin contents of pumpkin extracts. The third variable was assigned to be constant at the intermediate point while surface plots of three-dimensions were made by two independent variables.

4 $$ \begin{array}{l}\mathrm{Ellagic}\ \mathrm{acid}=3+0.046*{X}_1-0.0225*{X}_2-0.0441*{X}_3+0.06*{X}_1{X}_2+0.044*{X}_1{X}_3-0.025*{X}_2\\ {}{X}_3-0.112*{X}_1^2+0.00174*{X}_2^2-0.066*{X}_3^2\end{array} $$

5 $$ \begin{array}{l}\mathrm{Myricetin}=2.96+0.046*{X}_1-0.022*{X}_2-0.0445*{X}_3+0.060*{X}_1{X}_2+0.043*{X}_1{X}_3-0.025*{X}_2\\ {}{X}_3-0.11*{X}_1^2+0.00574*{X}_2^2-0.062*{X}_3^2\end{array} $$

**Fig. 5:**
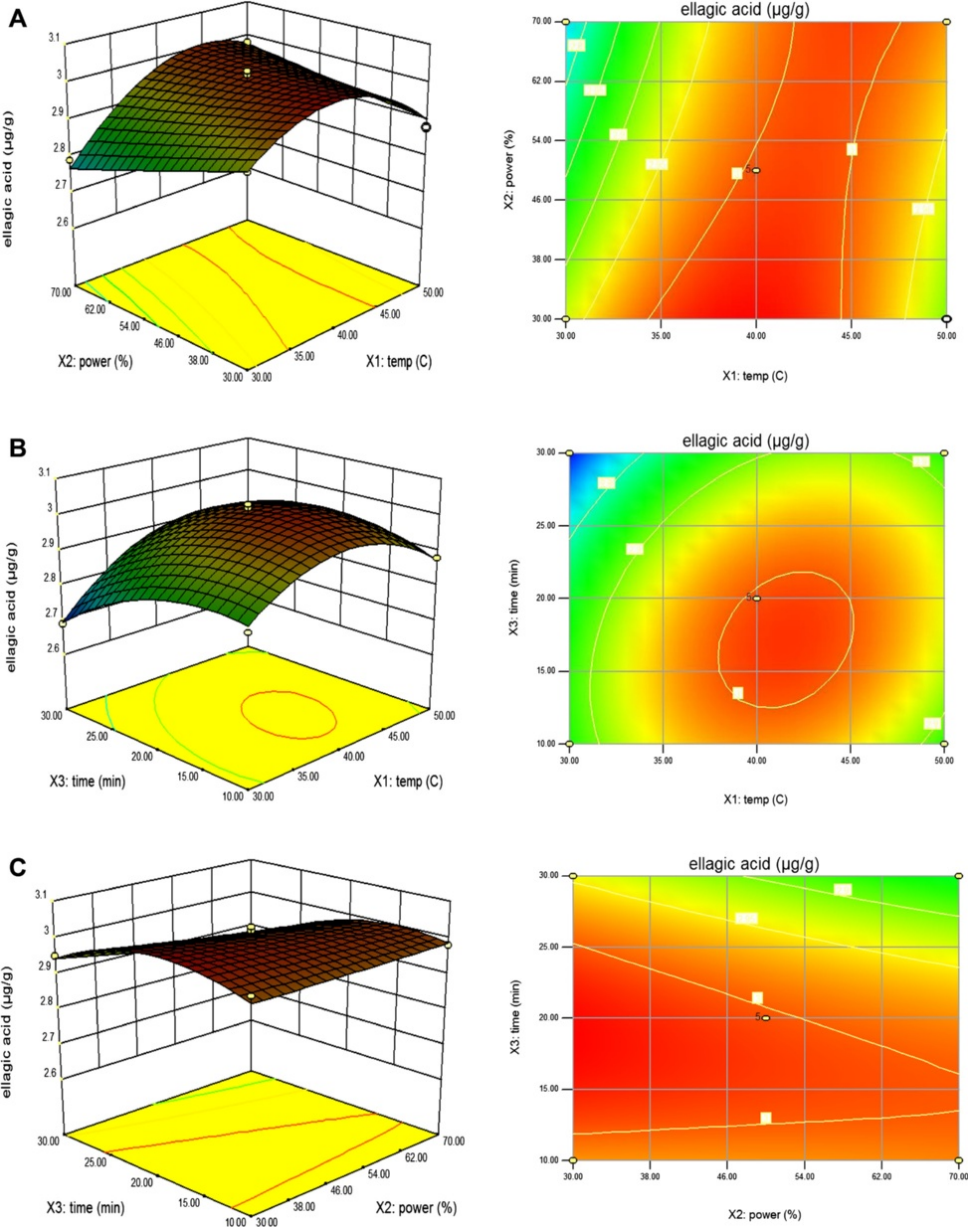
Response surface model plot showing the effects of of independent variables on ellagic acid content. Panel (**a**) temperature and power. Panel (**b**) temperature and time. Panel (**c**) power and time

As shown in Fig. 5a, when extraction time was fixed at 0 level, ellagic acid contents were improved while the extraction temperature increased from 30 °C to 38.81 °C, and reached the maximum value in the fixed extraction power of 33.23 %, and then the amount of ellagic acid contents decreased when the extraction temperature reached 50 °C due to the degradation of ellagic acid. The extraction amount of ellagic acid was affected by different ultrasonic extraction temperatures and ultrasonic extraction times as seen in Fig. 5b, when extraction power was fixed at 0 levels. It can be seen that the extraction amount of ellagic acid increased with the increasing ultrasonic extraction time and reached the maximum value at 18.51 min of extraction time. This finding was not in agreement with Zhang et al. [39] who found that the maximum value of ellagic acid from infructescence of *P. latycarya strobilacea L.* was at 40 min of extraction time. However, this result was concurred with Novak et al. [40] and Rostagno et al. [41] who confirmed that exposure to ultrasonic treatment for long time may cause loss to polyphenolic compounds due to denaturation, so it is very important to consider sonication time while processing. Figure 5c shows the effect of the interaction of extraction power and extraction time on the ellagic acid content at a fixed extraction temperature of 0 level. Maximum ellagic acid content was obtained at 18.51 min of extraction time in the fixed extraction power of 33.23 %. Moreover, the results were found that extraction temperature (X_1_) and extraction time (X_3_) were the most significant factor affecting the responses at the level of *p* < 0.01.

**Fig. 6:**
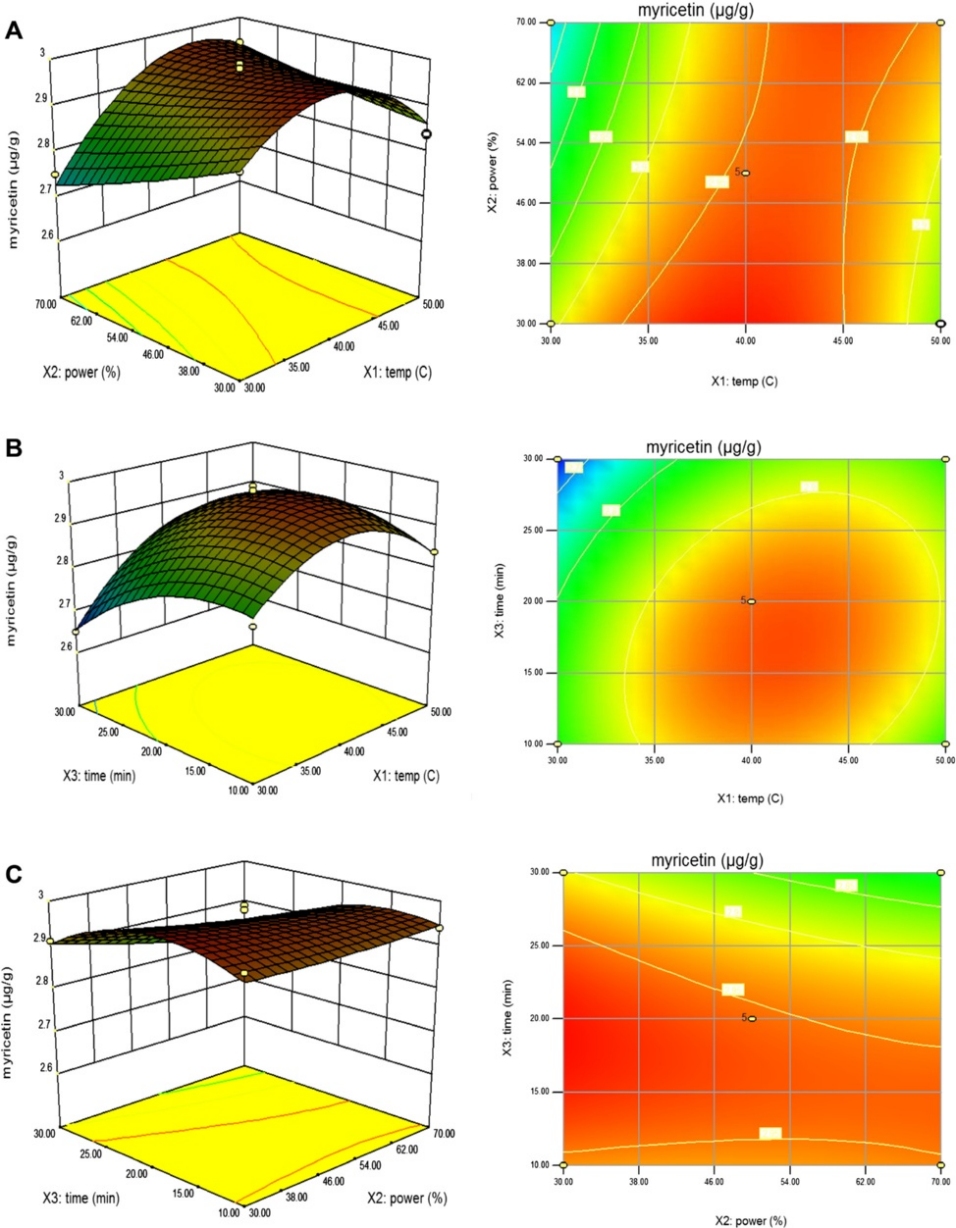
Response surface model plot showing the effects of of independent variables on myricetin content. Panel (**a**) temperature and power. Panel (**b**) temperature and time. Panel (**c**) power and time

Figure 6a shows the effect of the interaction of extraction temperature and extraction power on the myricetin contents at a fixed extraction time of 0 level. Maximum myricetin content was obtained at the highest extraction temperature and reached the maximum value at 38.98 °C of extraction temperature in the fixed extraction power of 33.79 %. Figure 6b shows the effect of the interaction of extraction temperature and extraction time on the myricetin content at a fixed extraction power of 0 level. Maximum myricetin content was also obtained by increasing extraction temperature to 40 °C in the fixed extraction time of 18.13 min. These results were in agreement with Shakthi Deve et al. [42] who found that the longest extraction time for flavonoids may result in loss to the polyphenols due to oxidation process. The oxidized products can convert to insoluble form compounds thereby diffusion of the polyphenols will be inhibited. Figure 6c shows the effect of the interaction of extraction power and extraction time on the myricetin content at a fixed extraction temperature of 0 level. Maximum myricetin content was obtained at the highest extraction power and reached the maximum value at 33.79 % of extraction power in the fixed extraction time of 18.13 min. Moreover, the results found that extraction temperature (X_1_) and extraction time (X_3_) were the most significant factor affecting the responses at the level of *p* < 0.01.

### Optimization and verification of the model for ultrasonic parameters

In order to verify the rutin and quercetin contents simultaneously from peach extracts, there was one optimal extraction conditions, which was established to get the highest values: modifying the extraction temperature of 41.08 °C to 40 °C, extraction power of 53.09 % to 50 %, and extraction time of 23.68 min to 24 min. The results are shown in Table 4 and the amounts of rutin and quercetin contents respectively under the optimal predicted conditions and experimental conditions. There was significant difference (*p* > 0.05) between the experimental and predicted values. Thus, this modification was not appropriate to assign in order to optimize the process of rutin and quercetin contents from peach. It seems that it was required to use the same optimized values for temperature, power, and time in order to get highest values for both rutin and quercetin contents.

**Table 4:** Predicted and actual experimental values of rutin and quercetin (μg/g) from peach extracts; and ellagic acid and myricetin (μg/g) from pumpkin under the optimal extraction conditions

Selected plants	Isolated compounds	Extraction variables			Predicated values	Experimental values^a^
		X_1_ (°C)	*X*_2_ (%)	X_3_ (min)		
Peach	R-P	41.08	53.09	23.68	2.906 ± 0.039	2.816 ± 0.0305
	Q-P				2.785 ± 0.040	2.733 ± 0.0208
Pumpkin	E-PP	38.99	33.12	18.15	3.030 ± 0.032	2.96 ± 0.05
	M-PP				2.986 ± 0.030	2.953 ± 0.06

^a^Mean ± standard deviation (*n* = 3). (*R-P*) = Rutin-peach; (*Q-P*) = Quercetin-peach; (*E-PP*) = ellagic acid-pumpkin; (*M-PP*) = myricetin-pumpkin

In order to facilitate the extraction process for pumpkin extracts, the optimal condition was modified as follows: the extraction temperature of 38.99 °C to 40 °C, and extraction power of 33.12 % to 33 %, and extraction time of 18.15 min to 18 min. The results are shown in Table 4 and the amounts of ellagic acid and myricetin contents respectively under the optimal predicted conditions and experimental conditions. There was no significant difference (*p* > 0.05) between the experimental and predicted values. Hence, the models can be used to optimize the process of ellagic acid and myricetin contents from pumpkin.

### MALDI identification

**Fig. 7:**
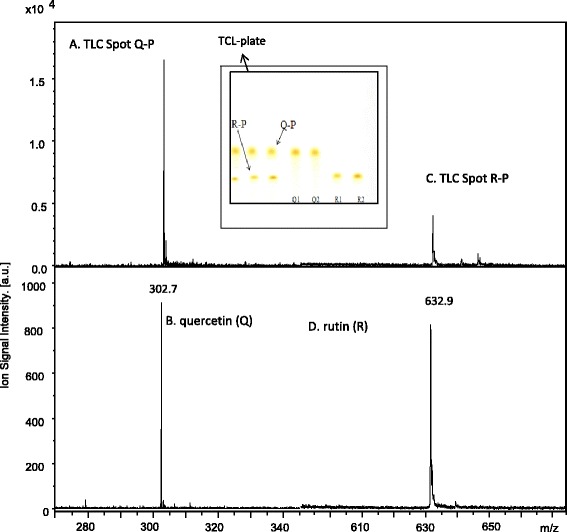
Mass spectra of peach extract TLC spot Q-P (**a**) and TLC spot R-P(**c**) excised and compared to quercetin Q (**b**) and rutin R (**d**) standards

Figure 7 shows the MALDI mass spectra obtained for TLC Spot Q-P (Fig. 7a) and TLC Spot R-P (Fig. 7c). Ion signals were observed at *m/z* 302.7 and 632.9. These ion signals were also observed in the MALDI mass spectra obtained from the certified quercetin (Q) (Fig. 7b) and rutin (R) (Fig. 7d) standards, respectively, and were assigned to the molecular radical cation (M^+.^) of quercetin (Q) and the sodiated cation (M + Na^+^) of rutin (R).

**Fig. 8:**
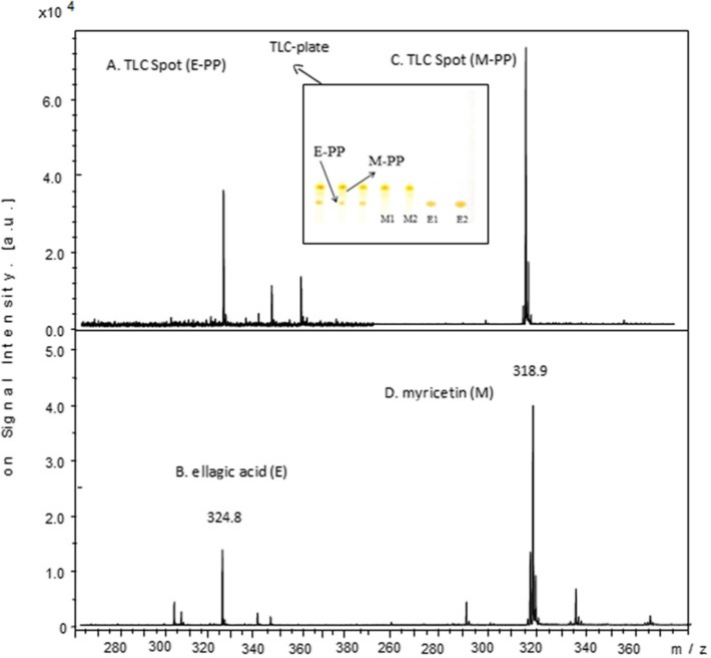
Mass spectra of pumpkin extract TLC spot E-PP (**a**) and TLC spot M-PP (**c**) excised and compared to ellagic acid E (**b**) and myricetin M (**d**) standards

Figure 8 shows the LDI mass spectrum obtained for the TLC Spot E-PP (Fig. 8a) and the MALDI mass spectrum obtained for the TLC Spot M-PP (Fig. 8c). Ion signals were observed at *m/z* 324.8 and 318.9, respectively, and were assigned to the sodiated cation (M + Na^+^) of ellagic acid (E) and molecular radical cation (M^+.^) of myricetin (M).

## Conclusion

The results of this study indicated that the ultrasonic treatments had the ability to enhance and increase the amount of polyphenol extraction yields from plants extracts (peach and pumpkin). TLC-densitometric method and BBD can be a very powerful technique in quantitative analysis of rutin and quercetin from peach extracts and ellagic acid and myricetin contents from pumpkin extracts. A high correlation of the quadratic polynomial mathematical model was gained and could be employed to optimize rutin and quercetin from peach extracts and ellagic acid and myricetin contents from pumpkin extracts by ultrasonic-assisted assay. The modified optimal extraction conditions for measuring rutin and quercetin simultaneously from peach extracts were as follows: extraction temperature of 41 °C, extraction power of 53 %, and extraction time of 24 min. Under these conditions, the experimental results of total rutin and quercetin contents were 2.816 ± 0.0305 μg/g of dry matter and 2.733 ± 0.0208 μg/g of dry matter respectively, which agreed closely with the predicted yield values. In contrast, the modified optimal extraction conditions for measuring ellagic acid and myricetin contents simultaneously from pumpkin extracts were as follows: extraction temperature of 40 °C, extraction power of 33 %, and extraction time of 18 min. Under these conditions, the experimental results of total ellagic acid and myricetin contents were 2.96 ± 0.05 μg/g of dry matter and 2.953 ± 0.06 μg/g of dry matter respectively, which agreed closely with the predicted yield values.
